# Radiosurgical treatment of solitary brain metastases using virtual cones with a standard multileaf collimator

**DOI:** 10.1002/acm2.13882

**Published:** 2022-12-28

**Authors:** Eric Lobb

**Affiliations:** ^1^ Department of Radiation Oncology Ascension NE Wisconsin‐St. Elizabeth Hospital Appleton Wisconsin USA

**Keywords:** stereotactic radiosurgery, virtual cone

## Abstract

**Purpose:**

The virtual cone has been previously introduced as a novel technique for generating small, spherical dose distributions using a high‐definition multileaf collimator (MLC) for functional radiosurgery applications. There has been no reported investigation into adapting this technique to a standard MLC for the treatment of solitary intracranial metastases as an alternative to physical stereotactic cones. This study characterizes the virtual cone technique adapted to a standard 5 mm leaf‐width MLC (VC_SD_).

**Methods:**

VC_SD_ dose distributions using MLC leaf gaps of 2–5 mm were generated and isodose sphericity metrics, peak dose gradients, optimal normalization ranges, and achievable field widths were compared to those of 5.0–12.5 mm diameter physical cones. Target sizes feasible to treat were identified and planned for comparison against established techniques using Paddick conformity index (PCI) and dose volume metrics. End‐to‐end validation of the VC_SD_ technique was performed.

**Results:**

VC_SD_ and physical cones sphericity metrics agree within 3.5% and VC_SD_ plans achieved a dose gradient of 21.3% mm^−1^, comparable to 10.0‐12.5 mm diameter physical cones. Normalization within the 50%–77% range preserves the optimal dose gradient within 2%⋅mm^−1^ and enables the treatment of 5–11 mm diameter planning target volumes (PTVs). Mean PCI for virtual and physical cones was 0.957 and 0.949, which compared favorably against conformal arc and VMAT (0.899 and 0.926). VC_SD_ outperformed conformal arc and VMAT for all dose volume metrics, and the mean 50% dose volume differed from physical cones by < 0.5cc for PTVs as small as 5 mm. Validation measurements showed 100% of points passing a 2% / 0.5 mm gamma test for all plans.

**Conclusions:**

The VC_SD_ technique efficiently generates spherical dose distributions for the treatment of small brain metastases. Characteristics of the VC_SD_ dose distributions are sufficiently comparable to those of physical cones to support VC_SD_ as an alternative for the treatment of spherical PTVs as small as 5 mm in diameter.

## 1 INTRODUCTION

The virtual cone, first introduced by Popple et al., is a novel multileaf collimator‐based field geometry technique used to create small, spherical dose distributions.^1^ The technique was introduced as an alternative to cone‐based collimation approaches for radiosurgical treatment of the trigeminal nerve and radiosurgical thalamotomy for the treatment of essential tremor, and has been implemented by at least one other reporting institution.^2^ As proposed, the virtual cone technique utilizes a high‐definition multileaf collimator with 2.5 mm leaf width, an MLC leaf gap fixed at the plan level, an externally‐developed and standardized control point sequence designed to generate a highly spherical dose distribution, and an MLC model within the treatment planning system specific to the virtual cone technique.

This initial implementation of the technique was reported to produce spherical dose distributions with comparable features to those of 4 and 5 mm diameter physical stereotactic cones, particularly when comparing volumes of the 10% dose cloud and above. The virtual cone technique was also reported to offer considerable efficiency benefits due to fast, templated application to specific patients, avoidance of the need for patient‐specific quality assurance measurements, and efficient MLC‐based treatment delivery.

Currently there has been no reported investigation into the potential adaptation of this technique to a standard multileaf collimation system with 5 mm leaf width. Although the coarser leaf width of a standard MLC would preclude the use of an adapted virtual cone technique for functional applications such as the treatment of trigeminal neuralgia, there may be an intermediate sized group of radiosurgical targets for which the technique could be used to improve the quality of MLC‐based treatment plans.

Specifically, leveraging the virtual cone technique may allow a subset of standard MLC‐based plans to mimic the superior dosimetric characteristics of physical stereotactic cones, narrowing the plan quality gap for small, spherical target volumes without requiring institutional investment in additional hardware, software, and associated maintenance. For institutions with low‐complexity radiosurgery programs where this potentially replaceable subset represents the smallest possible radiosurgical targets, this could provide an opportunity for cost and complexity reduction through consolidation of the program under exclusively MLC‐based planning approaches with a single treatment planning platform and radiosurgery dose calculation algorithm.

In this study the qualities of virtual cone dose distributions when adapted to a standard 5 mm leaf‐width multileaf collimator (VC_SD_) are evaluated and compared to those of physical stereotactic conical collimators with diameters in the range of 5.0–12.5 mm. Specifically investigated are dose distribution sphericity, achievable rates of dose falloff, and optimal normalization methods. Target sizes which are feasible to treat using the VC_SD_ technique are identified and planned using multiple alternative techniques in order to quantify dosimetric benefits and trade‐offs. Validation of a commercial treatment planning system's dose calculation output is performed for a range of VC_SD_ collimation techniques using film‐based end‐to‐end tests.

## 2 METHODS

### 2.1 Beam model and treatment platform

For VC_SD_ radiosurgical planning, all dose calculations utilized the AcurosXB 15.5.11 algorithm within the Varian Eclipse treatment planning system (Varian Medical Systems, Palo Alto, CA, USA), and exclusively utilized the 10‐MV flattening filter‐free (FFF) photon beam energy. All calculations used a 1 mm dose calculation grid with heterogeneity corrections enabled and dose reported to medium. The treatment platform was a Varian Truebeam (v2.7) with a standard multileaf collimation (MLC) system with 5 mm leaf width. Parameters within the Eclipse beam model including effective target spot size, MLC transmission, and MLC dosimetric leaf gap (DLG) have been tuned for stereotactic applications, though not specifically for the VC_SD_ technique. Beam data was acquired and validated down to a minimum jaw‐defined field size of 1 × 1 cm as part of the commissioning process. This AcurosXB model was also utilized for conformal arc and volumetric modulated arc therapy planning in this study.

For physical stereotactic cone planning, all calculations were performed using Brainlab iPlan 4.5 (Brainlab AG, Munich, Germany) with the pencil beam algorithm for conical collimators. All plans utilized the 10‐MV FFF photon energy with heterogeneity corrections enabled and a 1 mm isotropic dose calculation grid.

Treatment plans generated for this study were all created on a 1 mm slice‐thickness CT dataset of the CIRS Model 038 anthropomorphic head phantom (Computerized Imaging Reference Systems, Norfolk, VA, USA).

### 2.2 Virtual cone and physical cone dose distributions

Each VC_SD_ treatment plan consisted of an identical set of 10 treatment fields: a pair of full 360‐degree arcs at a table angle of 0 degrees and pairs of 180‐degree partial arcs at table angles of 36, 72, 288, and 324 degrees (IEC61217 scale). At each table angle, the field pair had collimator rotations of 45 and 315 degrees. X‐jaw and y‐jaw collimation in all fields were set to 1.0 and 1.5 cm, respectively, and the central two MLC leaves were opened to a static leaf‐gap while all other leaves were in a static closed position outside of the jaw‐defined aperture. Within a given plan the MLC leaf‐gap was fixed at a single value, and plans were generated that used leaf‐gap values of 2, 3, 4, and 5 mm.

As introduced by Popple et al.^1^, the virtual cone technique utilizes a custom control point sequence for each arc with the dose per degree proportional to the sine of the gantry angle. For a 2π irradiation, this creates a uniform fluence within the irradiated volume and results in a spherical dose distribution. Without this control point sequence there is unacceptable oblateness of the dose distribution in the anterior‐posterior direction.

A similar approach was utilized in this study for generation of the VC_SD_ treatment plans using a two‐degree control point spacing. Initial dose with uniform meterset progression was calculated using AcurosXB and the derived field‐specific meterset weight at control point *n* (*M_n_*) was computed according to the following:

$$\;M_{n}=\frac{D_{c,n}}{D_{c,n_{max}}}\;$$

where *D_c,n_* is the mean cumulative dose at control point *n* weighted proportional to the sine of that control point's gantry angle (*g_n_*), with averaged dose values *D_j,45_* and *D_j,315_* at collimator angles of 45 and 315 degrees, respectively, summed over all *j* control points for *j ≤ n*, as shown below:

$$\;D_{c,n}=\sum_{j=1}^{n}\frac{2*\left|\sin\left(g_{j}\right)\right|}{\left(D_{j,45}+D_{j,315}\right)}\;$$

Averaging the arc‐specific dose values at each control point for the 45‐degree and 315‐degree collimator settings facilitated the derivation of a control‐point sequence that is specific to only the table angle rather than specific to the combination of both table and collimator angle. The mean ratio of calculated dose for 45‐degree and 315‐degree collimator rotations across all control points was 1.000 ± 0.015, indicating that this averaging had negligible impact on the final calculated dose distribution.

**FIGURE 1:**
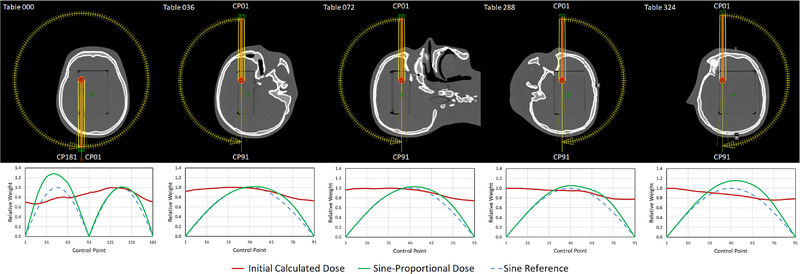
Example derivation of the field‐specific control point sequence for an off‐center target with asymmetric control‐point depths. The initial dose calculated using a uniform meterset progression is normalized to the reference sine curve to achieve the proportionally weighted dose, which is subsequently used to calculate the progressive weighting of each control point

An example control‐point sequence derivation for an off‐center target is provided in Figure 1, where the mean value of *D_j,45_* and *D_j,315_* normalized to the per‐field maximum is plotted as a function of control point number alongside the field's arc‐plane view. Normalizing the initially calculated dose (red) to a reference sine curve (blue) yields the sine‐proportional dose from which the meterset will be extracted.

**FIGURE 2:**
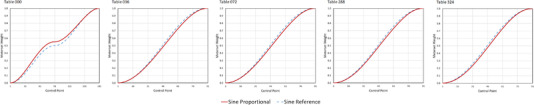
Target‐specific meterset progression for each table angle utilized with the virtual cone technique. The red curve is the dose‐corrected sine‐proportional solution shown in Figure 1, while the blue curve is directly weighted to the sine of the gantry angle without consideration of calculated dose, provided for reference only

**FIGURE 3:**
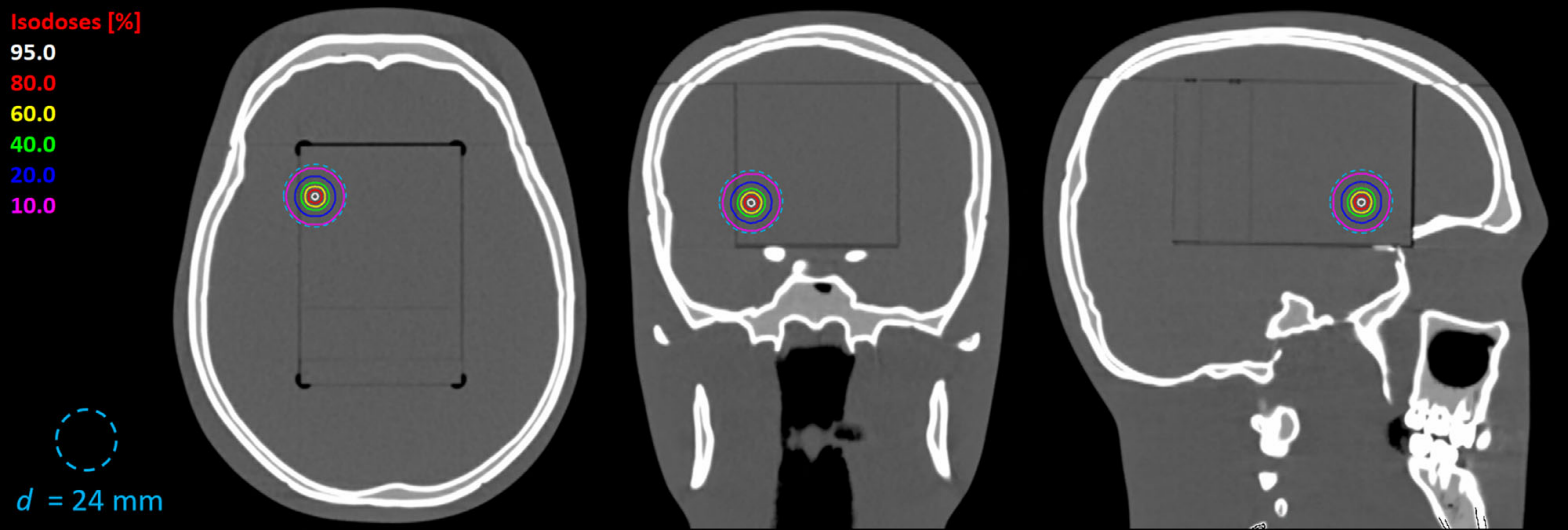
Example virtual cone dose distribution for the control‐point sequence from Figures 1 and 2. The blue circle is added for scale and is intentionally sized slightly larger than the 10% isodose line as a visual reference for the spherical quality of the dose distribution. Isodose percentages are relative to the isocenter dose value

Although plotted as a continuous function for clarity, the sine‐proportional goal dose is a discrete dataset with a resolution of one control point, corresponding to two degrees of gantry rotation. Summing all sine‐proportional dose points up to a given control point and then normalizing to the summation value of the entire dataset produces the meterset distribution for fields at each table angle (Figure 2). Also plotted in Figure 2 is a reference sine curve showing the meterset progression when the dose per control point is exactly fitted to the sine of the gantry angle (blue) rather than proportional to it (red) based on the initial uniform dose calculation. The difference between the reference and proportional curves at a given table angle is directly related to the degree of non‐uniformity in the effective depth of the treatment field. Inserting the derived control point sequence into the treatment fields for this target point results in the final dose distribution shown in Figure 3.

Dose distributions were also calculated for physical stereotactic cones with diameters 5.0, 7.5, 10.0 and 12.5 mm. Physical cone plans used the same set of table angles as the VC_SD_ plans, but arc lengths for partial arcs were reduced by 20 degrees from the poles to minimize fluence overlap in the anterior‐posterior direction. Additionally, each arc was subdivided into multiple weighted segments to achieve highly spherical dose distributions.

### 2.3 Dose distribution sphericity

Sphericity of all calculated dose distributions (virtual and physical cone) was determined for all integer isodose values in the range of 10%–80% of the isocenter dose, with isocenter positioned in the centroid of the “brain” of the CIRS Model 038 phantom. The minimum and maximum distances from isocenter to each isodose level were extracted from the 3D dose distribution using a custom DICOM RT‐Dose parser. The resulting maximum‐minimum distance ratio (“sphericity ratio”) was used to quantify sphericity for the VC_SD_ plans relative to the physical cone plans. A perfectly spherical dose distribution would therefore have a sphericity ratio value of unity, with the metric increasing linearly as the distribution becomes nonspherical.

### 2.4 Normalization strategies and treatable lesion sizes

The effect of normalization strategy on rate of dose falloff for all collimation techniques was determined by extracting the mean three‐dimensional perpendicular distance between each discrete isodose surface and the surface of its respective 50% isodose value. The mean falloff distance to 50% normalization dose was subsequently plotted as a function of normalization value to provide a visualization of how the dose gradient varies around the dosimetrically ideal value.

The width of the prescribed isodose surface is implicitly determined by the choice of normalization value. To determine the range of potential field widths available to a given MLC leaf gap, and therefore the range of target sizes possible to treat, the mean three‐dimensional width of each discrete isodose surface was extracted from each dose distribution. To facilitate visualization of target diameters possible to treat with a chosen combination of collimation technique and normalization value, the mean prescribed 3D field width was plotted as a function of normalization isodose value.

### 2.5 VC_SD_ plan comparisons with alternative techniques

To assess the practical differences between the VC_SD_ technique and other planning strategies in the context of potential clinical cases, a series of treatment plans were generated on the CIRS Model 038 CT dataset for centrally located spherical planning target volumes of diameter 5–11 mm. Plans for each target size were generated using the VC_SD_ technique, physical stereotactic cone planning, conformal arc planning, and VMAT planning. All plans were designed to deliver 2400 cGy in a single fraction to precisely 95% of the target volume using the same set of table angles and arc extents previously described for the VC_SD_ technique, except for the conformal arc and physical cone plans which included a 20‐degree gantry offset from the poles.

VC_SD_ plans were generated for the 5 and 6 mm diameter targets using a 2 mm leaf gap, for the 7 and 8 mm diameter targets using a 3 mm leaf gap, for the 9 mm diameter target using a 4 mm leaf gap, and for the 10 and 11 mm diameters targets using a 5 mm leaf gap. Physical stereotactic cone plans were generated for the 5 mm diameter target using a 5.0 mm diameter cone, the 6–7 mm diameter targets using a 7.5 mm diameter cone, the 8–10 mm diameter targets using a 10.0 mm diameter cone, and the 11 mm diameter target using a 12.5 mm diameter cone. Conformal arc plans were all generated using a 0 mm aperture margin on each respective target volume with collimator angles selected to generate the most conformal and spherical dose distribution achievable.

VMAT plans were generated using the Photon Optimizer 15.5.11 with structure resolution set to Fine, Convergence Optimization Mode set to On, Dose Calculation Resolution of the optimization engine set to High, and Intermediate Dose performed. Dose gradient was driven using the Normal Tissue Objective with a target border distance of 0 mm, a starting dose of 100%, an ending dose of 20%, and a fall‐off factor of 0.50. These parameters define a continuous gradient objective which aims to achieve a reduction to 50% and 25% of the prescribed dose within distances of 2.1 and 5.7 mm from the target surface, respectively. An additional ring structure with inner surface 12 mm from the target edge was used to constrain the spread of the 10% isodose volume. Lower objectives were used to drive target coverage without the use of upper objectives.

To compare techniques, the absolute volume of the 10%, 25%, and 50% isodose clouds external to each target volume (“dose spill volume”) was tabulated for each planning approach. Additionally, total plan monitor units for each plan and technique was recorded as the MU efficiency ratio, defined as the ratio of the total fraction MU and prescribed fraction dose. Conformity of the prescribed dose to each respective target volume was recorded using the conformity index definition proposed by Paddick (PCI)^3^:

$$PCI\;=\frac{{\left(TV_{PIV}\right)}^{2}}{TV\times PIV}$$

In the above equation for PCI, *TV* is the volume of the target structure, *PIV* is the volume of the prescription dose value, and *TV_PIV_* is the volume of the overlap between the prescription dose value and the target structure. PCI values range from 0 (indicating no overlap of the target volume and prescribed dose volume) to 1 (indicating perfect overlap).

### 2.6 Impact of target location on VCSD plan quality

Use of the VC_SD_ technique for generalized treatment of brain metastases requires that the technique be capable of generating high‐quality dose distributions for targets located essentially anywhere within the brain. To assess the extent to which plan quality is affected by target location, a group of 21 points representative of potential target centroids were distributed through the “brain” of the Model 038 phantom. Points were distributed in an approximately uniform lattice with a mean distance between points of 3.63±0.26 cm (median distance 3.70 cm), and a maximum distance from any point in the “brain” to its closest target point of 4.26 cm. VC_SD_ treatment plans were generated for 8 mm diameter target spheres centered at each of these 21 points, with location‐specific control‐point sequencing derived according to the previously described process. Each plan was normalized to achieve exactly 95% target coverage at 2400 cGy using a 3 mm leaf gap.

Paddick conformity index was recorded for each target as well as the volume of the 10%, 25%, and 50% dose spill volumes. Additionally, 3D sphericity ratios were tabulated for each treatment plan using the previously described methodology.

### 2.7 Field aperture verification

The 2D apertures of MLC‐defined fields with leaf gaps of 2 mm, 3 mm, 4 mm and 5 mm were initially validated using enface irradiation of Gafchromic EBT3 radiochromic film (Ashland Advanced Materials, Bridgewater, NJ, USA). Film was positioned between virtual water slabs (4 cm buildup, 10 cm backscatter) at 100 cm source‐to‐surface distance (SSD) and irradiated with sufficient monitor units to deliver approximately 350 cGy to the film center according to treatment planning system calculation (MU range of 550–700 depending on leaf gap).

Calibration for absolute dosimetry was achieved by irradiating a set of films from the same manufactured lot to 13 equally spaced known dose values in the range of 0–600 cGy via cross‐calibration against an ADCL‐calibrated Farmer‐type ionization chamber.

Irradiated films were digitized using an Epson 10000XL flatbed scanner (Epson America Inc., Long Beach CA) at 300 DPI and 48‐bit color depth. Data from the red channel was imported into RIT v6.9 (Radiological Imaging Technology Inc., Colorado Springs CO, USA) for creation of the dose calibration curve and conversion of the irradiated leaf‐gap films to absolute dose. Film handling, digitization timing, and consistency in irradiation and digitization orientation and positioning were all performed consistent with published recommendations.^4^ Film noise was reduced through application of a median smoothing filter of size 3 × 3 pixels.

Dose calculated by the Eclipse treatment planning system at the level of the film in the coronal plane was exported for comparison purposes. The calculated and measured dose planes were registered via mutual identification of dosimetric landmarks and normalized to the dose value of their respective centroid positions. Comparison profiles for each of the 4 discrete leaf gaps in the IEC‐X direction (leaf‐gap direction) were generated and evaluated using a 1D distance to agreement (DTA) analysis. The DTA analysis technique was selected and applied to the normalized dose profiles in order to evaluate the relative accuracy of the calculated leaf gap profile widths without the analysis being confounded by uncertainties in overall absolute dose scaling.

### 2.8 End‐to‐end treatment plan verification

Full 3D VC_SD_ treatment plans were generated on the CIRS Model 038 phantom using the 2 mm, 3 mm, 4 mm, and 5 mm leaf gap geometries. The plans were normalized such that 500 cGy was delivered to isocenter, with the isocenter being positioned at the geometric center of the coronal‐plane film holder (CIRS Model 038‐05) embedded in the phantom.

The Model 038 phantom was precisely positioned for plan delivery using a combination of surface guidance and CBCT imaging with six degree‐of‐freedom correction capabilities. Residual positioning errors of the phantom determined with verification CBCT imaging were ≤ 0.1 mm and ≤ 0.1 degrees. After inserting the film in the phantom, the treatment delivery was initiated and carried out to completion. According to surface monitoring, the magnitude of phantom displacement due to couch walkout was ≤0.5 mm and ≤0.2 degrees across the full range of couch positions.

Irradiated films were handled, digitized, and converted to absolute dose consistent with the previously described process. Registration of the film and exported DICOM dose plane was accomplished using mutual identification of dosimetric landmarks. Non‐normalized absolute dose comparison profiles for each plan were generated in the IEC‐X (right‐left) and IEC‐Y (superior‐inferior) directions. The resulting profiles were evaluated using a 1D gamma test with 2% dose and 0.5 mm distance criteria, with these analysis parameters selected to stringently evaluate the spatial alignment of the narrow dose profiles while allowing for a modest dosimetric uncertainty inherent in the film dosimetry process.

## 3 RESULTS

### 3.1 Dose distribution sphericity

**FIGURE 4:**
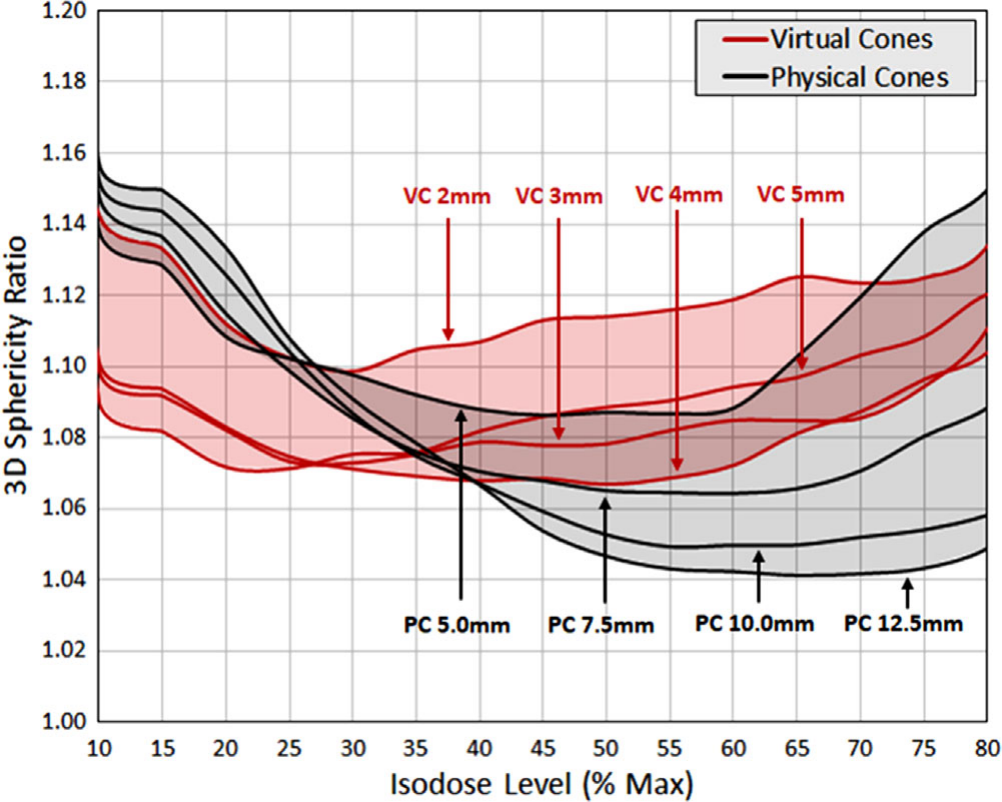
Sphericity ratio comparisons for MLC‐based virtual cones (“VC”) with leaf gaps of 2–5 mm (red) and physical stereotactic cones (“PC”) of diameter 5.0–12.5 mm (black) as a function of isodose value relative to dose maximum. The shaded regions encompass the full range of each respective technique to aid in overall trend comparisons. Lower values indicate more spherical isodose shells, with a minimum possible value of unity

Sphericity ratio results are presented in Figure 4 for relative isodose values from 10% to 80%. Each plotted line represents a single dose distribution calculated with either the VC_SD_ or physical stereotactic cone technique, and the shaded bands encompass all distributions calculated with each respective technique in order to better visualize the overall trends.

Mean sphericity ratios for the group of VC_SD_ plans are within approximately 3.5% of mean sphericity ratios for the group of physical cone plans in the 10%–80% isodose surface range. When averaging sphericity ratios across all plans and isodose values, the ratio of VC_SD_ to physical cone results was 1.005 ± 0.024.

### 3.2 Strategies and treatable lesion sizes

**FIGURE 5:**
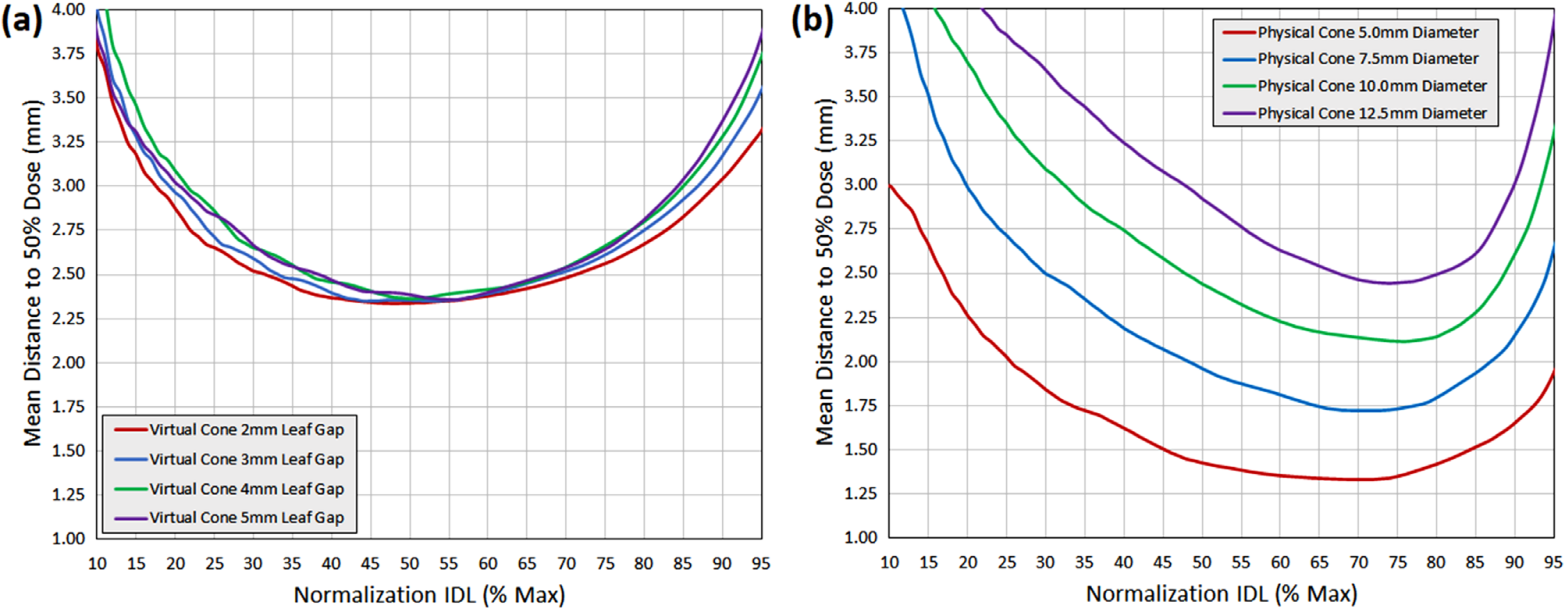
Mean 3D distance to 50% of the normalization isodose value as a function of normalization isodose value for (a) VCSD and (b) physical stereotactic cone treatment plans

Plots of mean 3D distance to 50% of normalization dose for all leaf‐gap plans are provided in Figure 5. The normalization isodose value that results in the sharpest dose falloff for all VC_SD_ plans is in the range of 48%–56%, while for plans generated with physical cones with diameters 5.0–12.5 mm the optimal value is in the range of 70%–76%. Peak achievable three‐dimensional rate of dose falloff for all VC_SD_ plans was approximately 21.3%⋅mm^−1^, which falls between what is achievable for the 10 mm and 12.5 mm physical cones (23.7%⋅mm^−1^ and 20.4%⋅mm^−1^, respectively), and is less rapid than what is achievable using the 5.0‐ and 7.5‐mm physical cones (37.6 mm^−1^ and 28.9 mm^−1^, respectively).

A noteworthy property of the VC_SD_ plans is the relatively flat shape of the dose falloff distribution, indicating a uniform rate of dose falloff across a broad range of isodose values. For VC_SD_ plans, normalization within the range of 30%–77% results in the same mean 3D distance to half‐dose within approximately 0.25 mm, which corresponds to a maximum reduction in dose falloff rate of approximately 2%⋅mm^−1^ relative to the optimal value.

**FIGURE 6:**
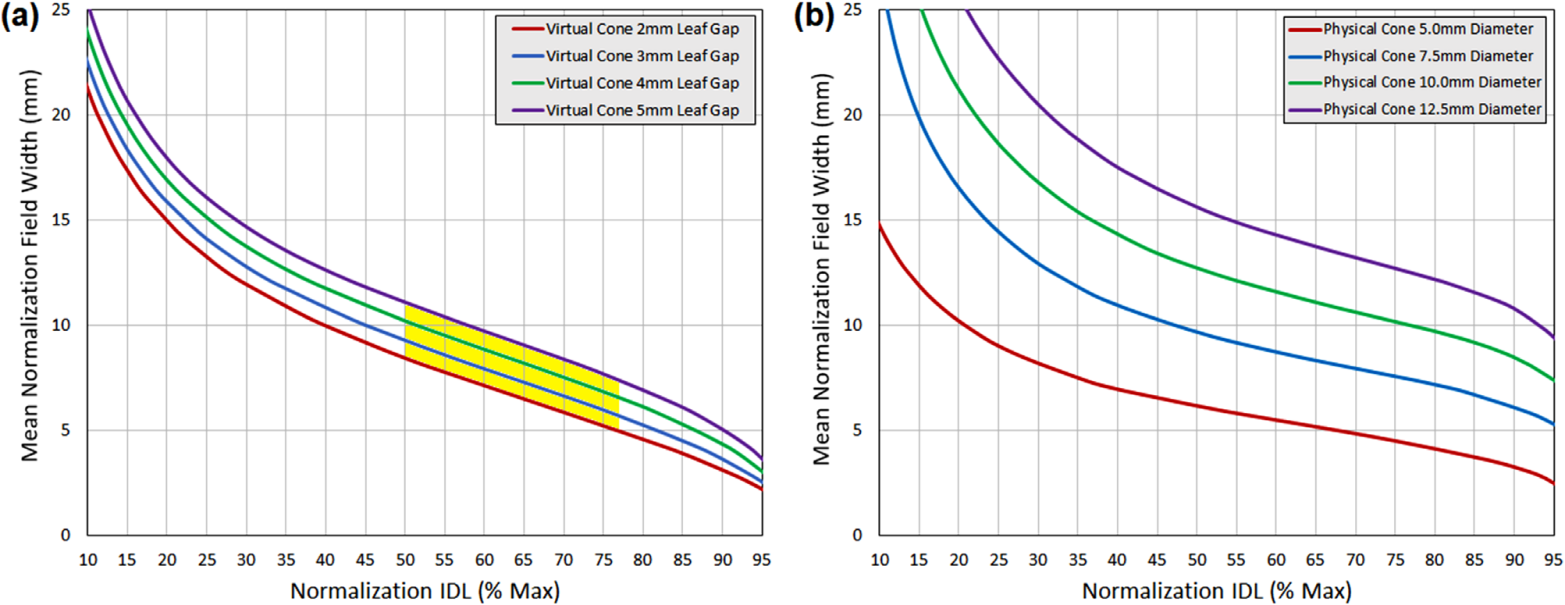
Mean 3D field width at the normalization isodose value as a function of normalization isodose value for (a) VC_SD_ and (b) physical stereotactic cone treatment plans. The shaded region in (a) represents the normalization options and associated field widths that achieve a dose gradient within 2%⋅mm^−1^ of the optimal value

Mean 3D field width at the normalization isodose level is plotted for all VC_SD_ plans as a function of normalization isodose level in Figure 6, with data for physical cones also provided for comparison purposes. In combination with the normalization value selection data previously discussed, this field width data provides information on the range of target sizes that can be treated with the VC_SD_ technique while retaining near‐optimal rates of dose falloff. If it is desired to keep the rate of dose falloff within 2%⋅mm^−1^ of the optimal value then normalization within the 50%–77% range would be acceptable (disregarding options < 50%), corresponding to a field diameter range and approximate treatable target diameter range of 5–11 mm.

### 3.3 VC_SD_ plan comparisons with alternative techniques

**FIGURE 7:**
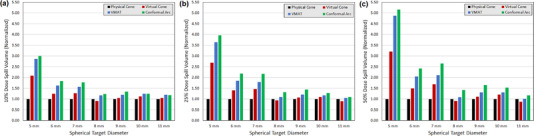
Comparison histograms of (a) 10%, (b) 25%, and (c) 50% isodose volume external to the target volume for various planning techniques. Values at each data point are normalized to the physical stereotactic cone value

Comparison histograms of 10%, 25%, and 50% dose spill volumes (which exclude the volume of the target) for each plan type are provided in Figure 7. Due to the wide range of absolute volumes reported, values are normalized to the physical stereotactic cone results to facilitate simplified comparison. Physical cones had the lowest dose spill volume for 16 of the 21 data points, with VC_SD_ plans having the lowest volumes for the remaining 5 data points. In all cases VC_SD_ outperformed both conformal arc and VMAT planning techniques for this set of spherical targets.

There is a marked difference in data spread when stratifying the results into three groups: the 5 mm diameter target planned with a 5 mm diameter physical cone, the 6–7 mm diameter targets planned with a 7.5 mm diameter physical cone, and the 8–11 mm diameter targets planned with the 10.0 mm and 12.5 mm diameter physical cones. Mean 10%, 25%, and 50% absolute dose spill volumes for all techniques and stratified into these three size groups is provided in Table 1.

**TABLE 1:** Mean absolute dose spill volumes stratified into target diameter groups of 5 mm, 6–7 mm, and ≥8 mm

	10% dose spill volume (cc)			25% dose spill volume (cc)			50% dose spill volume (cc)		
Technique	5 mm Target	6–7 mm Target	8–11 mm Target	5 mm Target	6–7 mm Target	8–11 mm Target	5 mm Target	6–7 mm Target	8–11 mm Target
Physical cones	3.39 ± 0.00	7.07 ± 0.58	15.61 ± 3.16	0.66 ± 0.00	1.49 ± 0.09	3.74 ± 0.96	0.16 ± 0.00	0.40 ± 0.01	1.02 ± 0.27
Virtual cones	7.07 ± 0.00	8.86 ± 0.79	16.19 ± 3.88	1.77 ± 0.00	2.14 ± 0.18	3.69 ± 0.78	0.51 ± 0.00	0.63 ± 0.05	1.02 ± 0.19
VMAT	9.70 ± 0.00	11.28 ± 0.70	18.74 ± 3.91	2.39 ± 0.00	2.71 ± 0.12	4.18 ± 0.88	0.77 ± 0.00	0.83 ± 0.03	1.17 ± 0.21
Conformal Arc	10.18 ± 0.00	12.74 ± 0.80	19.40 ± 3.32	2.60 ± 0.00	3.23 ± 0.20	4.70 ± 0.74	0.82 ± 0.00	1.01 ± 0.06	1.43 ± 0.20

Isodose percentages are relative to the target margin dose of 2400 cGy.

As expected based on peak achievable dose gradient data, the VC_SD_ technique could not match the dose spill volumes of the 5.0 mm and 7.5 mm physical cones. Compared to the 5.0 mm physical cone, the VC_SD_ plans had a mean increase in dose spill volume of approximately 270%. When compared to plans using the 7.5 mm diameter physical cone, this mean increase dropped to 45%. Finally, for target diameters ≥ 8 mm planned with either the 10.0 or 12.5 mm diameter physical cones, the mean dose volume spill increase for the VC_SD_ plans was 2.5%.

However, the absolute difference between VC_SD_ and physical cone plans is also important to consider when working with targets of this size. The excess dose spill volume of the 50% isodose cloud (1200 cGy) for the VC_SD_ plans compared to the 5.0 mm diameter physical cone was 0.4 cc, and the excess volume of the 10% isodose cloud (240 cGy) was 3.9 cc. When compared to the 7.5 mm diameter physical cone these excess volumes were 0.2 cc and 1.9 cc, respectively. These values dropped to < 0.1 cc and < 1 cc, respectively, for target diameters ≥ 8 mm.

**FIGURE 8:**
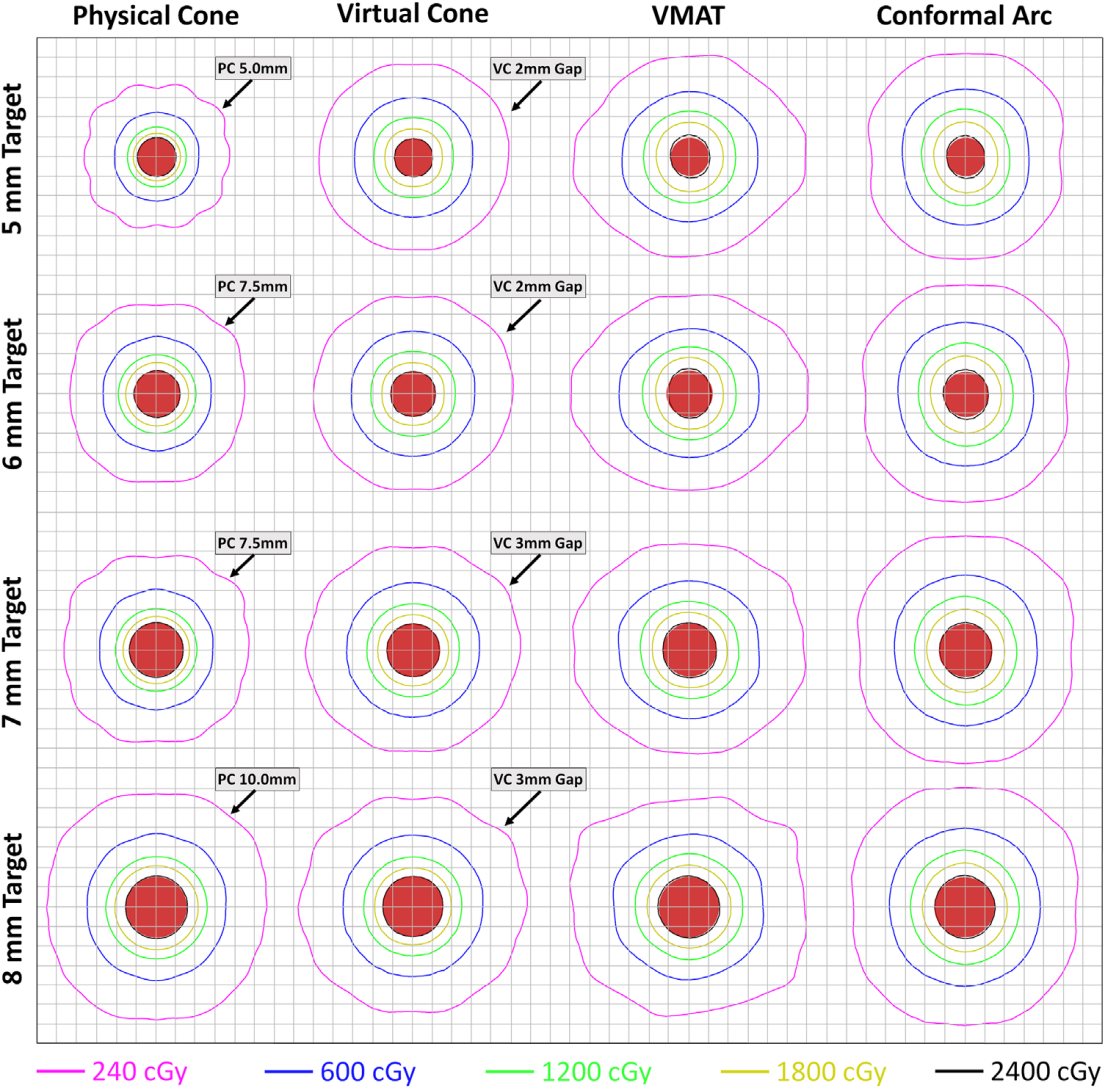
Mean isodose distributions for each evaluated technique and planning target diameters of 5–8 mm. Shown distributions are the composite average through the primary anatomical planes. All plans are normalized such that 2400 cGy covers 95% of the PTV. Each grid square is sized 2.5 × 2.5 mm and the red circles are 2D projections of the spherical target volumes. PC, Physical Cone; VC, Virtual Cone

Figure 8 provides example technique‐specific isodose distributions for planning target volume diameters of 5–7 mm—where the benefit of physical cones is greatest—as well as 8 mm, where the techniques begin to converge in quality. Each of the provided isodose distributions represents the mean composited distribution through the primary anatomical planes.

**FIGURE 9:**
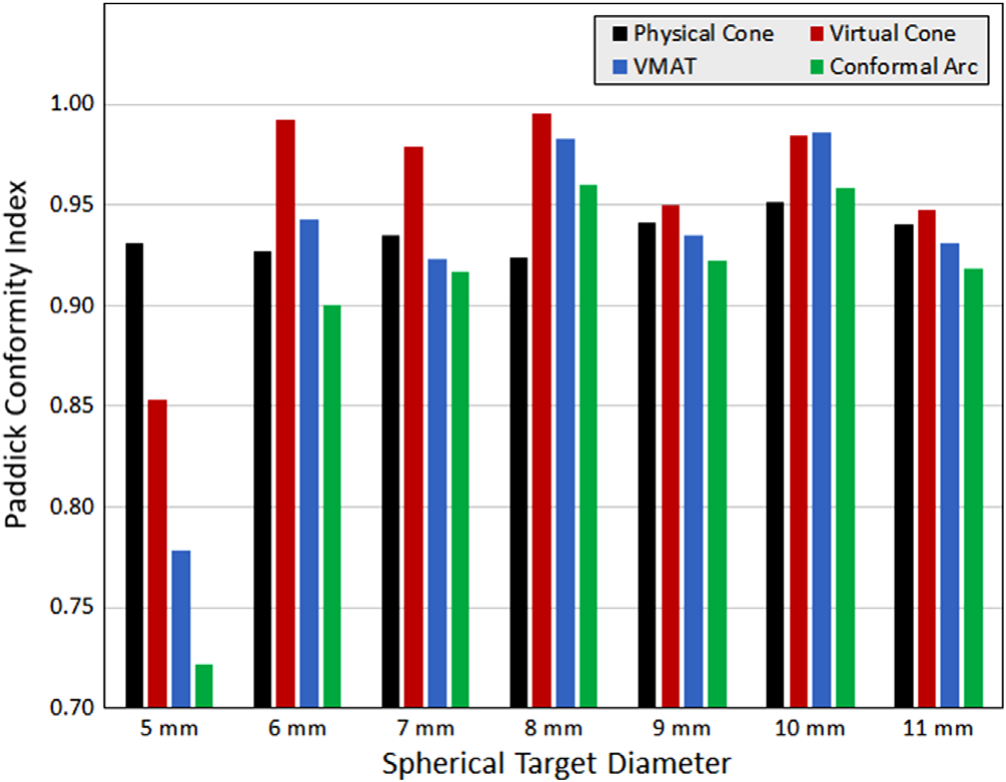
Paddick conformity index values (non‐normalized) for various planning strategies

Results for Paddick conformity index are provided in Figure 9. For this set of spherical targets, the VC_SD_ technique had the highest average conformity index (0.957), followed by physical stereotactic cones (0.936), VMAT (0.926), and conformal arc (0.899). The 5 mm diameter target was the greatest conformity stratifier with physical cones achieving a value of 0.931, followed by 0.853 for VC_SD_, 0.778 for VMAT, and 0.722 for conformal arcs. Conformity index variability reduced substantially starting at a target diameter of 6 mm and remained relatively consistent between techniques for larger targets (all values ≥ 0.900). VMAT underperforming in dose conformity can be explained by the fact that the studied targets were spherical and both the VC_SD_ and physical cone techniques are purpose‐designed to generate matching spherical dose distributions in a 2π irradiation geometry, whereas VMAT relies on the modulation of a small number of coarse MLC leaves to shape the dose distribution.

The VC_SD_ technique required approximately 50% more monitor units than other non‐modulated techniques, with a mean MU efficiency ratio across all plans of 3.08 ± 0.18 compared to 1.96 ± 0.37 for physical cones and 2.02 ± 0.08 for conformal arcs. The MU efficiency of the VC_SD_ technique was comparable overall to VMAT, which had an MU efficiency ratio of 3.38 ± 0.94. The heightened MU requirement of the VC_SD_ technique is explained by the consistently small field aperture and lower average plan normalization, which had a mean value of 64.3 ± 8.1% across all target sizes.

### 3.4 Impact of target location on VC_SD_ plan quality

Table 2 provides 10%, 25%, and 50% dose spill volumes for targets positioned throughout the Model 038 “brain”, as well as Paddick conformity index values and mean 3D sphericity ratios with values averaged across the 10%–80% relative dose range. There was 0.6 cc maximum variation in the volume of the 10% dose spill volume (range of 10.7–11.3 cc), 0.1 cc variation in the 25% dose spill volume (range of 2.59–2.69 cc), and < 0.1 cc variation in the 50% dose spill volume (range of 0.74–0.76 cc). Paddick conformity index values for each of the plans varied within the range 0.92–0.97. Achievable dose gradient did not vary appreciably with varying intracranial location, with mean peak dose gradient across the 21 target locations of 21.04 ± 0.13%⋅mm^−1^. Mean sphericity ratios within the 10%–80% isodose range were 1.07–1.09.

**TABLE 2:** Recorded metrics for targets with variable intracranial location, including dose spill volumes (DSV), Paddick conformity index values, and mean sphericity ratios averaged over the 10%–80% relative dose range

Target number	10% DSV (cc)	25% DSV (cc)	50% DSV (cc)	Conformity index (PCI)	Mean sphericity ratio (10%–80%)
01	10.96	2.66	0.76	0.97	1.08 ± 0.01
02	10.74	2.59	0.74	0.92	1.08 ± 0.01
03	10.80	2.59	0.74	0.94	1.08 ± 0.01
04	10.82	2.59	0.74	0.94	1.08 ± 0.01
05	10.82	2.59	0.74	0.94	1.08 ± 0.01
06	11.05	2.63	0.75	0.95	1.08 ± 0.01
07	10.78	2.59	0.74	0.94	1.08 ± 0.01
08	11.02	2.62	0.75	0.94	1.09 ± 0.01
09	10.88	2.61	0.75	0.93	1.09 ± 0.01
10	10.86	2.61	0.75	0.93	1.08 ± 0.01
11	10.98	2.63	0.75	0.92	1.08 ± 0.01
12	11.06	2.64	0.75	0.93	1.08 ± 0.01
13	11.15	2.65	0.76	0.94	1.08 ± 0.01
14	11.12	2.64	0.75	0.93	1.08 ± 0.01
15	10.95	2.62	0.75	0.93	1.09 ± 0.01
16	11.18	2.66	0.75	0.94	1.08 ± 0.01
17	11.05	2.64	0.75	0.93	1.09 ± 0.01
18	11.20	2.66	0.76	0.94	1.08 ± 0.01
19	10.99	2.62	0.75	0.94	1.08 ± 0.01
20	11.34	2.69	0.76	0.94	1.07 ± 0.01
21	11.32	2.69	0.76	0.94	1.09 ± 0.01
**Mean**:	**11.00** ± **0.17**	**2.63** ± **0.03**	**0.75** ± **0.01**	**0.94** ± **0.01**	**1.08** ± **0.00**

Multi‐plane visualization of each intracranial target location is provided in Supplement A.

The uniformity of these metrics across all evaluated potential target positions indicates that the VC_SD_ technique can be applied to metastases throughout the brain without dosimetric compromise. Field‐specific control point sequences and sphericity ratio plots for each of the 21 evaluated target locations are provided in Supplement A, as well as multi‐plane visualization of each target position.

### 3.5 Field aperture verification

**FIGURE 10:**
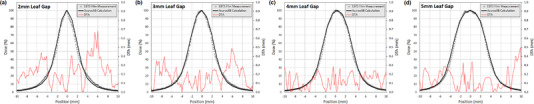
Comparison of AcurosXB calculated profiles and measured profiles using EBT3 radiochromic film for static enface VC_SD_ treatment fields. Relative dose is displayed on the left vertical axis while distance to agreement (DTA) value is plotted in red and displayed on the right vertical axis. Profiles are shown in the direction of MLC travel (IEC‐X, leaf‐gap direction) for (a) 2 mm, (b) 3 mm, (c) 4 mm, and (d) 5 mm MLC leaf gaps

Comparisons of measured and calculated profiles (normalized to the maximum dose value) in the leaf‐gap direction are provided for leaf gaps of 2, 3, 4, and 5 mm in Figure 10. Also included in each figure is a plot of 1D DTA. Mean DTA values for calculated points ≥ 10% of the centroid dose were 0.22 ± 0.13 mm, 0.15 ± 0.08 mm, 0.13 ± 0.07 mm, and 0.13 ± 0.09 mm for leaf gaps of 2, 3, 4, and 5 mm, respectively. The maximum DTA values for these leaf gaps were 0.49, 0.32, 0.29, and 0.34 mm, respectively.

### 3.6 End‐to‐end treatment plan verification

**FIGURE 11:**
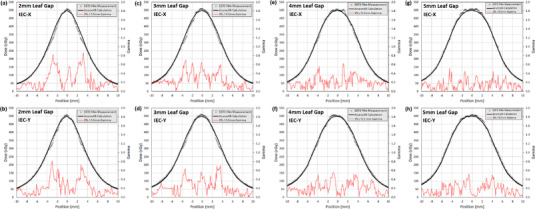
Comparison of AcurosXB calculated profiles and measured profiles using EBT3 radiochromic film for full end‐to‐end tests of the VC_SD_ technique in the CIRS Model 038 phantom. Absolute dose in cGy is displayed on the left vertical axis while 2% / 0.5 mm gamma value is plotted in red and displayed on the right vertical axis. Profiles are shown in both the IEC‐X (left‐right) and IEC‐Y (superior‐inferior) directions for (a,b) 2 mm, (c,d) 3 mm, (e,f) 4 mm, and (g,h) 5 mm MLC leaf gaps

Comparisons of measured and calculated absolute dose profiles in both the IEC‐X (left‐right) and IEC‐Y (superior‐inferior) directions for full VC_SD_ treatment plans utilizing a 2, 3, 4, and 5 mm leaf gap are shown in Figure 11, along with an overlay of gamma comparison values using a 2% dose and 0.5 mm distance criteria. For each measured profile the gamma passing rate was 100%. The maximum per‐field gamma value ranged from 0.52 to 0.85 and the mean gamma values for points > 10% of the maximum calculated dose ranged from 0.17 to 0.24.

## 4 DISCUSSION

The virtual cone technique adapted to a standard multileaf collimator with 5 mm isocentric leaf width (VC_SD_) and MLC leaf gaps of 2–5 mm has been evaluated for the radiosurgical treatment of small brain metastases. Mean overall sphericity metrics for the VC_SD_ technique dose distributions are within 1% of those for physical stereotactic cones, and peak dose falloff rates for the VC_SD_ technique were found to approximate what is achievable with 10 mm and 12.5 mm diameter physical cones. Target diameters that can be treated with the VC_SD_ technique while keeping the rate of dose falloff within 2%⋅mm^−1^ of the optimal value were found to be 5–11 mm, requiring plan normalization within the 50%–77% range. While a normalization value near 50% for MLC‐based radiosurgery is likely lower than utilized in many practices, the 50%–75% range has been suggested as potentially optimal for cranial radiosurgery by Zhao et al.^5^ Clinicians accustomed to radiosurgery planning with maximum central dose values in the approximate range of 130%–150% of the prescribed dose (67%–77% normalization) can continue that practice with the VC_SD_ technique.

Although the VC_SD_ technique could not match the dose gradient of the 5.0 and 7.5 mm diameter stereotactic cones used for treatment of spherical targets < 8 mm in diameter, the absolute difference was minor with mean excess 1200 cGy dose spill volumes of ≤0.5 cc for a 2400 cGy prescription. For spherical targets ≥8 mm in diameter the dose gradient of the VC_SD_ technique converged to that of the 10 and 12.5 mm diameter physical cones within a mean difference of 2.5%. Further, the VC_SD_ technique achieved dosimetrically favorable plan quality over alternative MLC‐based techniques such as conformal arc and volumetric modulated arc therapy for all spherical targets, and the quality of the calculated VC_SD_ treatment plan did not depend on the target's location within the brain.

This study characterized VC_SD_ plans for leaf gaps in discrete millimeter increments, but users of the Eclipse treatment planning system can specify the leaf gap directly in increments of one‐tenth of a millimeter. Considering the relative constancy in achievable dose gradient across broad normalization ranges for leaf gaps from 2 to 5 mm, it is unlikely that such sub‐millimeter optimizations would result in a substantial improvement in plan‐specific dose gradient or normal tissue sparing within this range.

The VC_SD_ technique also provides an efficient treatment planning workflow once a set of plan templates has been built. The appropriate template can be applied directly to a new clinical case, calculated and normalized to the desired target coverage, and the result will be a predictably spherical dose distribution with a rapid and approximately isotropic rate of dose falloff.

The results of the validation measurements performed in this study suggest that our institution's existing small‐field beam model is sufficiently accurate for use with the VC_SD_ technique. However, this cannot be assumed and implementation of the VC_SD_ technique requires appropriate institution‐specific commissioning and validation. In addition to the film‐based aperture and end‐to‐end testing provided here, both the original virtual cone paper by Popple et al. and the independent implementation paper by Brown et al. provide information on the use of commercial plastic scintillating detectors for output verification and detector‐based end‐to‐end measurements, as well as the use of parallel‐plate detectors for tracking virtual cone output constancy over time. Additionally, Brezovich et al. have described a custom phantom designed for radiosurgery accuracy testing that has been applied to small MLC‐defined fields using static leaf gaps as small as 1.6 mm.^6^

Some consideration of the delivery efficiency of the VC_SD_ technique is warranted. Although the 10 MV FFF photon beam energy is capable of dose rates up to 2400 MU per minute, the large number of arcs and resulting division of total plan monitor units results in a plan delivery that becomes limited by the gantry rotation speed. The time required to complete the delivery of a 2400 cGy VC_SD_ treatment plan after verification imaging was consistently measured at 9.8 min during validation tests, and this included the use of efficient field ordering and remote table rotations without room entry between fields. It is well documented that prolonged radiosurgery treatment times result in increased patient position deviation ^7^, ^8^, and appropriate motion monitoring and mitigation strategies therefore are critical. Use of the 6 MV FFF photon beam energy, which is capable of dose rates up to 1400 MU per minute on a Varian Truebeam, would result in modestly reduced dose spill volumes without the loss in delivery efficiency that would occur with a treatment plan that is dose‐rate limited. In the publication introducing the virtual cone technique, Popple et al. reported approximately 10% reductions in V25% values and 25% reductions in V10% values for 6 MV FFF compared to 10 MV FFF with a high‐definition multileaf collimator.^1^

As described, this technique is only suitable for the treatment of solitary targets where the isocenter is aligned with the target centroid, as with physical stereotactic cones. The treatment of multiple brain metastases with the virtual cone technique would require sequential targeting and re‐positioning of the patient between the delivery of each plan, and the dosimetric quality of the resulting plan and compromises in treatment delivery efficiency would have to be weighed against established multi‐met treatment techniques such as those utilizing a single isocenter with simultaneous targeting of all lesions.

Existing radiosurgery programs with standard multileaf collimators that also utilize physical stereotactic cones for the treatment of spherical planning target volumes no smaller than 5 mm (which may be a 3 mm diameter lesion with a 1 mm uniform planning margin) could potentially consolidate their program to exclusively use MLC‐based techniques by implementing the VC_SD_ technique. New radiosurgery programs built around a standard MLC that anticipate a primary focus on the treatment of brain metastases without a functional radiosurgery component could also benefit from having the VC_SD_ technique available as a tool. In either case, the VC_SD_ technique could provide a qualifying program with opportunities for reductions in the numbers of managed hardware and software systems, dose calculation algorithms, and clinical workflows.

Implementation of the virtual cone technique requires a custom control point sequence to drive sphericity of the dose distribution. Previous publications on the virtual cone technique have referenced the use of prestored control point sequences and plan‐generation scripts for application to specific patients.^1^, ^2^ Currently, institutions looking to implement the virtual cone technique would either need sufficient resources and expertise to develop the control point sequence themselves or would need to rely on inter‐institutional data sharing. Direct integration of this technique into the treatment planning system by the manufacturer would provide the most accessible solution, and potentially allow for more robust utilization through algorithmic selection of optimal static leaf gaps and control‐point meterset weighting for a specific target size and intracranial location.

## 5 CONCLUSIONS

The virtual cone technique adapted to a standard multileaf collimator with 5 mm leaf width (VC_SD_) is feasible to utilize for efficient generation of spherical dose distributions for the radiosurgical treatment of small brain metastases. Relevant characteristics of the VC_SD_ dose distributions are sufficiently comparable to those of physical stereotactic cones to support VC_SD_ as an alternative for the treatment of spherical planning target volumes as small as 5 mm in diameter, with favorable plan quality over alternative MLC‐based techniques such as conformal arc and volumetric modulated arc therapy. While physical stereotactic cones can achieve superior peak dose gradients for target diameters ≤ 7 mm when using 5.0‐ and 7.5‐mm diameter cones, the reduction in volume of the resulting 50% isodose cloud external to the target was found to be < 0.5 cc when compared to the VC_SD_ technique. Institutions with relatively low‐complexity radiosurgery programs could utilize the VC_SD_ technique as an alternative to physical stereotactic cones as a strategy for cost reduction, complexity reduction, or technology consolidation under an exclusively MLC‐based program.

## AUTHOR CONTRIBUTION

Study design and execution as well as drafting of the manuscript was performed by the listed author.

## CONFLICT OF INTEREST

None.

## Supporting information



Supplementary Information
